# Characterization of Albendazole-Randomly Methylated-β-Cyclodextrin Inclusion Complex and *In Vivo* Evaluation of Its Antihelmitic Activity in a Murine Model of Trichinellosis

**DOI:** 10.1371/journal.pone.0113296

**Published:** 2014-11-18

**Authors:** Agustina García, Darío Leonardi, María D. Vasconi, Lucila I. Hinrichsen, María C. Lamas

**Affiliations:** 1 Departamento de Farmacia, Universidad Nacional de Rosario, Rosario, Santa Fe, Argentina; 2 IQUIR-CONICET, Universidad Nacional de Rosario, Rosario, Santa Fe, Argentina; 3 Área Parasitología, Facultad de Ciencias Bioquímicas y Farmacéuticas, Universidad Nacional de Rosario, Rosario, Santa Fe, Argentina; 4 Instituto de Genética Experimental, Universidad Nacional de Rosario, Rosario, Santa Fe, Argentina; 5 CIC-UNR, Facultad de Ciencias Médicas, Universidad Nacional de Rosario, Rosario, Santa Fe, Argentina; Hungarian Academy of Sciences, Hungary

## Abstract

Albendazole is a benzimidazole carbamate extensively used in oral chemotherapy against intestinal parasites, due to its broad spectrum activity, good tolerance and low cost. However, the drug has the disadvantage of poor bioavailability due to its very low solubility in water; as a consequence, a very active area of research focuses on the development of new pharmaceutical formulations to increase its solubility, dissolution rate, and bioavailability. The primary objective of this study was to prepare randomly methylated β-cyclodextrins inclusion complexes to increase albendazole dissolution rate, in order to enhance its antiparasitic activity. This formulation therapeutic efficacy was contrasted with that of the pure drug by treating *Trichinella spiralis* infected mice during the intestinal phase of the parasite cycle, on days five and six post-infection. This protocol significantly decreased muscle larval burden measured in the parenteral stage on day 30 post-infection, when compared with the untreated control. Thus, it could be demonstrated that the inclusion complexes improve the *in vivo* therapeutic activity of albendazole.

## Introduction

Trichinellosis is a foodborne parasitic zoonosis widely distributed all over the world in most climates, except for deserts, with a burden of approximately 10,000 people per year and a 0.2% mortality rate. [1] Humans acquire the infection by ingestion of undercooked meat contaminated with *Trichinella* spp. Muscle encysted larvae are set free after gastric digestion and maturing into adult worms that penetrate the mucosa of the intestine. After fertilization, female worms release newborn larvae that disseminate throughout the host to find the striated muscle where they encyst [1]–[3]. The administration of efficacious anthelmintic drugs at the stage of intestinal invasion is remarkably important to obtain an effectual therapy [4].

Albendazole (ABZ) is a benzimidazole carbamate widely used for the treatment of trichinellosis [1]–[4]. The drug belongs to Class II in the Biopharmaceutics Classification System. It is poorly water soluble and highly lipophilic (log *P* of 2.55) and, consequently, it can exhibit unfavourable bioavailability after oral administration, leading to variable oral absorption [5]. In this context, several formulation techniques have been previously investigated to increase ABZ dissolution rate, including solid dispersion, polymeric microencapsulation and complexation with cyclodextrins (CDs) [5]–[12].

The use of CDs as suitable pharmaceutical excipients is an attractive alternative to prepare liquid and solid formulations to enhance the dissolution rate and bioavailability of poorly soluble drugs [13]–[15].

Accordingly, β-CD has attained pharmaceutical relevance despite its low aqueous solubility, since chemical modifications increase this parameter due to the loss of the crystalline solid state [16]–[18]. Furthermore, Lajos Szente and Jozsef Szejtli [19], postulated that the random substitution of any hydroxyl group produces an interference of the stable hydrogen bond system around the β-CD rims causing an intensive enhancement of the aqueous solubility. Thus, several CD derivatives of pharmaceutical interest have been developed including the hydroxypropyl derivatives of β-CD and γ-CD, the randomly methylated β-CD (RM-β-CD), sulfobutylether β-CD, and the so-called branched CDs such as maltosyl-β-CD [20]. Hydroxypropyl β-CD (HP-β-CD) is much more water-soluble and more toxicologically benign than the natural β-CD, and its most important use is for the intravenous route.

There is an extensive prior work on the use of CDs to improve ABZ water solubility. Including others authors, Bassani et al., [9] proposed a liquid formulation which included ABZ in HP-β-CD, in a molar ratio of 1/10, which resulted in a significant increase in the drug aqueous solubility, up to 3500 times, compared with the native β-CD to be administered by the parenteral route.

Concerning the oral route, Diaz et al. [21], studied inclusion complexes employing α, β, γ and HP-CD to improve the solubility behavior and ABZ dissolution rate. The complexation has been studied by using electronic absorption spectroscopy and molecular mechanics. In addition, Castillo et al. [7] formulated ABZ:HP-β-CD freeze-dried inclusion complexes. They observed an important increase of the ABZ solubility, although a supersaturation effect was observed when drug release studies were performed in non-sink conditions.

The role of RM-β-CDs in improving oral drug delivery and dissolution rate is based on the dramatic rise in the aqueous solubility of the RM-β-CDs (>500 mg/mL) (18.5 mg/mL) [22] and the affinity for the hydrophobic molecules in comparison with the β-CD. RM-β-CDs are absorbed moderately from the gastrointestinal tract into the systemic circulation, enhancing the oral bioavailability and drug permeability by making the drug accessible at the surface of the biological barrier [13], [19], [23], [24]. Furthermore, RM-β-CD may also inhibit P-gp function in small intestinal epithelial cells after oral administration, resulting in an increase in the drug activity [24]. Another important use of RM-β-CD is to protect some molecules, such as proteins, from gastrointestinal conditions. Sajaresh et al. proposed an interesting oral insulin delivery system based on RM-β-CD complexed insulin encapsulated in polymethacrylic acid (PMAA) hydrogel microparticles evaluated under *invitro* conditions [23].

This work aimed to modify the ABZ water solubility through complexation with RM-β-CD. The inclusion complex and physical mixtures were prepared and methodically characterized using ultraviolet–visible (UV–vis) spectroscopy, X-ray diffraction, differential scanning calorimetry, and nuclear magnetic resonance spectroscopy (^1^H-^13^C and Rotating-Frame Overhauser Effect Spectroscopy, ROESY) and dissolution studies. The stoichiometry and equilibrium constants, which allow quantitative description of the solubility behavior, were determined by phase-solubility studies.

The antiparasitic activity of the ABZ:RM-β-CD complex was evaluated in a murine model of trichinellosis [25], [26]. ABZ and the inclusion complex formulations were administered during the intestinal phase of the parasite cycle, on days 5 and 6 post-infection.

This study has been focused on providing an efficient approach in order to develop ABZ pharmaceutical formulations for oral administration, with high water solubility and antiparasitic activity.

## Materials and Methods

### Materials

ABZ (1 µg/mL aqueous solubility), β-CD, HP-β-CD, RM-β-CD were supplied by Sigma-Aldrich Chemie GmbH (Steinheim, Germany). All other chemicals were of analytical grade.

### Methods

#### Solubility diagrams

Solubility diagrams were obtained according to Higuchi and Connors [27]. Briefly, excess amounts of ABZ (30 mg) were added to 10 mL of water containing various concentrations (0–60 mM) of β-CD, HP-β-CD or RM-β-CD. Samples were shaken for 72 h in an elliptical shaker at 180 rpm and 37°C and then filtered through a 0.45 µm membrane filter (Advantec MFS, Inc.). ABZ concentration in the filtrate was spectrophotometrically analyzed at 291 nm (Boeco S-26 spectrometer, Hamburg, Germany). The phase-solubility diagrams were obtained and the complex formation constant (*K*f) were calculated according to the equation:

S (tan α) S_0_ (solubility of the drug in the absence of CD).
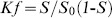



Each experiment was performed in triplicate.

#### Preparation of inclusion complexes and physical mixtures

The inclusion complexes were prepared by spray drying. ABZ was dissolved in acetic acid; water and β-CD, HP-β-CD or RM-β-CD, were added to that solution. The molar ratio was 1∶1. The resulting solutions were spray-dried in a Mini Spray Dryer Buchi B-290 (Flawil, Switzerland) under the following conditions: inlet temperature, 130°C; outlet temperature, 70°C; air flow, 38 m^3^/h; feed, 5 mL/min; aspirator set, 100%.

Additionally, physical mixtures between ABZ and β-CD or RM-β-CD were prepared in a mortar by mixing the drug and carrier for 10 minutes.

### Complex Characterization

#### Differential scanning calorimetry

Differential scanning calorimetry (DSC) was performed on a Shimadzu TA-60 (Kyoto, Japan) calorimeter, using 5 mg samples in crimped aluminium pans. The instrument was calibrated with indium and zinc as standards. Nitrogen was used as a purge gas, and an empty aluminium pan was employed as a reference. Each sample was scanned at a rate of 5°C/min from 25 to 350°C, under N_2_ atmosphere (flow rate 30 mL/min).

#### X-Ray Diffraction

Data collection was carried out in transmission mode on an automated X’Pert Phillips MPD diffractometer (Eindhoven, The Netherlands). X-ray diffraction patterns were recorded using CuKα radiation (λ = 1.540562 Å), 40 kV voltage, 20 mA current and steps of 0.02° on the interval 2*θ* = 10°–40°. Low peak broadening and background were assured by using parallel beam geometry with an X-ray lens and a graphite monochromator placed before the detector window. Data acquisition and evaluation were performed with the Stoe Visual-Xpow package, Version 2.75 (Germany).

#### ESI-MS analysis

The stoichiometry of the complex was confirmed by mass spectrometry with high resolution. The equipment used was a micrOTOF-Q II spectrometer, Bruker-Daltonics (Bremen, Germany) equipped with an electrospray ionization source (ESI). ESI-MS conditions were optimized to improve the analysis of the complexes. The spectrometer parameters were set as follows: ESI (negative ion mode), collision energy: −10 eV, spray voltage 2.8 kV, nebulizer: 0.4 bar, dry heater: 180°C, drying gas flow rate: 4.0 L/min, end plate offset: −500 V, set collision Cell RF: 300.0 Vpp. The spectra of the investigated compounds were acquired in the range m/z 50–3000. ABZ:RM-β-CD inclusion complex was dissolved in a 0.4% v/v solution of formic acid (FA).

#### ROESY experiments

Two-dimensional rotating-frame Overhauser effect spectroscopy (ROESY) experiments were used to confirm the current complexation of ABZ with RM-β-CD, as well as to characterize their binding mode. The ABZ:RM-β-CD complex (10 mg) was solubilized in 0.5 mL 0.1 N DCl in D_2_O, and filtrated (0.45 µm Millipore membrane filter). ROESY measurements were performed with a Bruker Avance 300 instrument (Karlsruhe, Germany) with a 5 mm probe using the roesyph pulse sequence (Bruker) with the experimental conditions as follows: 32 scans, acquisition time 0.222 s, pulse delay 1.92 s and 512 data points [28]. Resonance at 4.7 ppm was used as an internal reference due to residual solvent water (H_2_O and HDO).

#### Dissolution studies

Dissolution studies were performed in 900 mL 0.1 N HCl at 37°C, according to the U.S. Pharmacopeia [29] Apparatus 2 (SR8 8-Flask Bath, Hanson Research, Chatsworth, CA) with paddle rotating at 50 rpm. Samples of ABZ pure drug, physical mixtures or spray-dried complexes equivalent to 100 mg of the drug were spread on the surface of the dissolution medium and the time 0 was recorded. At appropriate time intervals, 5 mL samples were withdrawn and filtered (pore size 0.45 mm). The amount of drug released was determined by UV analysis, measuring the absorbance spectrophotometrically at 291 nm.

Dissolution efficiency (DE) a concept proposed by Khan and Rookes in 1975 [30] and defined as the area under a dissolution curve between specified time points, was calculated using the following equation:
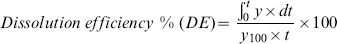
where y is the percentage of dissolved product at time t.

#### Antiparasitic activity assay in *Trichinella spiralis* infected mice

Susceptible CBi+ mice, of the CBi colony from the Animal Facilities of the Instituto de Genética Experimental, Facultad de Ciencias Médicas, Universidad Nacional de Rosario (from here on, CBi-IGE stock), were used to evaluate the *in vivo* effect of ABZ formulations on *Trichinella spiralis* muscle cyst formation. Mice and facilities conditions were described elsewhere [6]. Briefly, young adults were housed, separated by sex since weaning, in polypropylene cages (32 cm×24 cm×10 cm) with wood shavings for bedding, in a room with constant temperature (24±2°C), with a relative humidity of 50±10% and a light-dark illumination cycle of 12 h. Cages and bedding were changed twice a week. Tap water and food (Cargill Laboratory Chow, pelletized) were provided *ad libitum*. Adult CBi+ males (90–100 days old) were weighed (mean weight 54±3 g) and orally infected with 2 *Trichinella spiralis* infective muscle larvae per g of body weight. The larvae used in the infection were recovered by artificial digestion in a solution of 1% pepsin 1% HCl at 37°C, from the muscles of a CBi+ mouse that had been infected 2–3 months earlier; the infection dose for each animal was prepared by counting individual larvae. After infection, the animals were divided in three groups (n = 5 per group) to test the effect of the solid systems on *Trichinella spiralis* infectivity: I) ABZ; II) ABZ:RM-β-CD complex and III) infection control without treatment. Groups I and II were treated with 50 mg ABZ per kg of body weight on days 5 and 6 post-infection.

During the course of the experience, the animals were examined three times a week to determine whether infection altered their physical condition, looking for signs and symptoms of trichinellosis. No signs of illness, pain or distress were observed [31].

The anthelmintic activity of the novel ABZ formulation was assessed on the chronic phase of the infection (32±2 days post-infection), by counting the number of muscle encysted parasites (muscle parasitic burden) in the tongue of each mouse, as already described [6]. Briefly, after euthanizing the mouse, by inhalation of CO_2_, the tongue was excised, weighed and digested in 2 mL of digestion fluid. The mixture was incubated at 37 °C overnight and stirred periodically at moderate speed. At the end of the incubation period, digestion was stopped by adding saline to complete 14 mL and the larvae were allowed to decant for 1 h. The supernatant was removed immediately and 5 mL of 10% v/v formalin were admixed in order to inactivate and preserve intact the structure of the larvae. Finally, all larvae on each sample were counted under a microscope with a 40X magnification. Parasite burden was calculated as the total number of larvae per g of muscle tissue (PBr). The reduction percentage of PBr in the treated animals was calculated as follows:
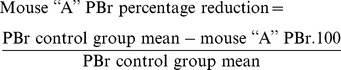



All the experiments with mice were done during the first half of the light cycle and according to animal care standards of the institution, which complies with guidelines established by the Institute for Laboratory Animal Resources. The protocol was approved by the Animal Care and Use Committee of the National University of Rosario (Permit Number: 648/2012).

The statistical significance of the differences in PBr among groups was assessed by the non-parametric Kruskal-Wallis analysis of variance, and Dunn’s post-test was used for comparisons between groups. Variables expressed as percentages were studied with the Mann-Whitney “U” test. Differences were considered significant if *p*<0.05.

## Results and Discussion

### Solubility diagrams of the ABZ:CDs formulations

The complex formation constants (*K*f) showed the relationship between ABZ and the corresponding CDs (β-CD, HP-β-CD and RM-β-CD). The phase solubility diagrams of HP-β-CD and RM-β-CD fit an A_L_-type profile very well [27]. This curve indicates the formation of soluble 1∶1 type inclusion complex. The *K*f value obtained for the inclusion complex between ABZ and RM-β-CD was 336.70 M^−1^ suggesting a stable complex in comparison with the *K*f obtained with β-CD: 67.90 M^−1^ and HP-β-CD: 313.16 M^−1^. Thus, according to the solubility isotherms obtained, ABZ improved its solubility with RM-β-CD in comparison with β-CD and HP-β-CD.

The better *K*f value obtained for RM-β-CD supported the selection of this derivative to formulate the inclusion complexes, complete its characterization and evaluate *in vivo* its anthelmintic activity.

### Characterization of the ABZ:RM-β-CD complexes and physical mixtures

#### Differential scanning calorimetry

**Figure 1:**
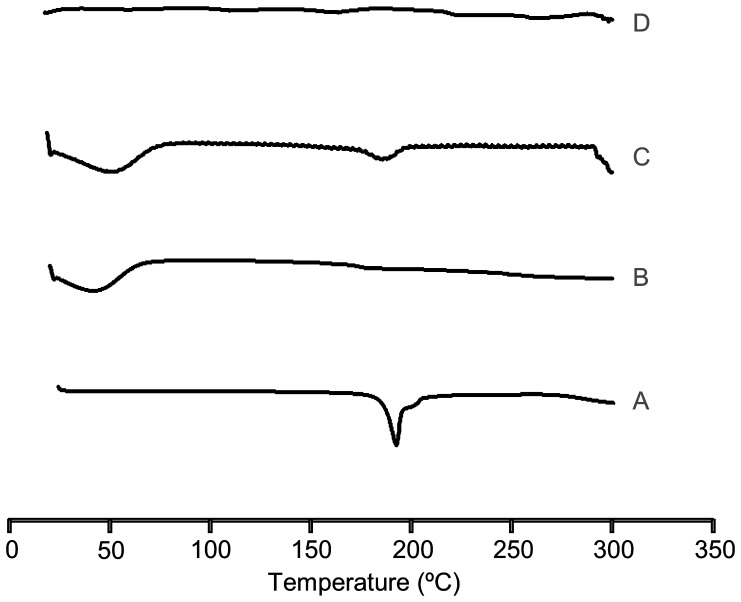
DSC thermograms. A: ABZ (pure drug), B: RM-β-CD, C: ABZ:RM-β-CD obtained by physical mixtures, D: ABZ:RM-β-CD obtained by spray drying.

Figure 1 shows DSC thermograms of ABZ, RM-β-CD and the systems prepared by physical mixtures and spray drying. The characteristic sharp melting peak of ABZ was observed at 196.84 °C. The intensity of the drug endothermic peak was also recorded in the physical mixture system thermogram. The absence of the ABZ peak in the spray dried system could be due to the formation of an inclusion complex [32], [33].

#### X-Ray Diffraction

**Figure 2:**
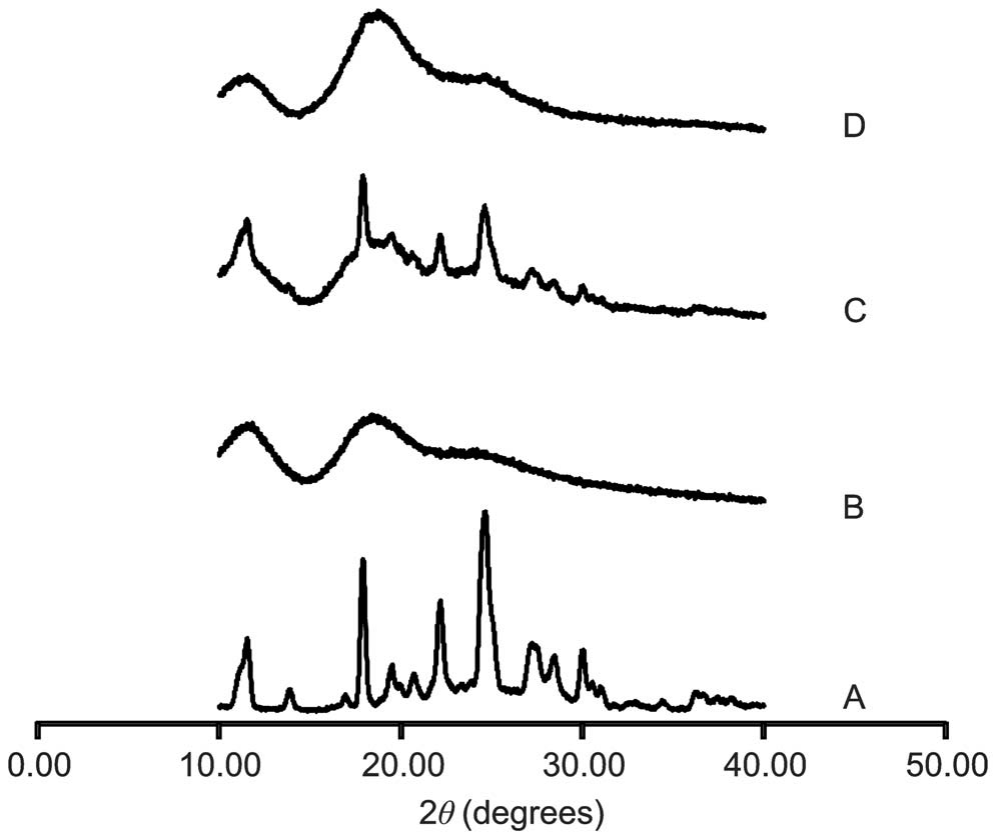
X ray diffraction patterns. A: ABZ (pure drug), B: RM-β-CD, C: ABZ:RM-β-CD obtained by physical mixtures, D: ABZ:RM-β-CD obtained by spray drying.

The powder X-ray patterns of ABZ, RM-β-CD and ABZ:RM-β-CD systems obtained by physical mixtures and spray drying are shown in Figure 2. The derivative RM-β-CD showed an amorphous structure; ABZ presented intense and characteristic diffraction peaks at 2*θ* 11.51; 17.85; 22.09 y 24.54. The diffraction patterns of ABZ in the physical mixtures indicated the simple overlapping of the ABZ and RM-β-CD diffraction peaks, indicating that the crystallinity of ABZ did not change essentially. The lower intensity of some ABZ peaks could be a dilution effect due to the presence of the RM-β-CD. On the other hand, the diffraction patterns of the spray drying system showed wider intense peaks, implying that ABZ is in an amorphous state. XRD analyses are in substantial agreement with DSC data. The thermal parameters of ABZ:RM-β-CD complex could be attributed to partial complexation of the drug in the CD cavity, confirming the amorphizing role of RM-β-CD [34].

#### ESI-MS analysis

**Figure 3:**
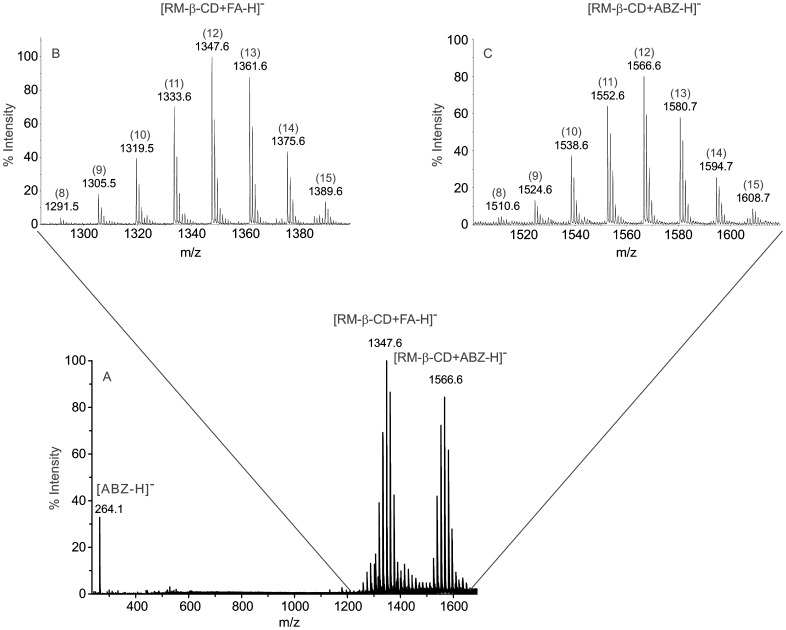
ESI-MS spectrum. A: Partial MS spectrum of ABZ:RM-β-CD obtained by spray drying dissolved in a formic acid solution. B and C: Zoom of regions corresponding to RM-β-CD and ABZ:RM-β-CD, respectively. The masses and the number of methyl substituents are written above each peak.

Figure 3 shows a part of the MS spectrum of ABZ:RM-β-CD (spray dried) dissolved in FA solution. The multiple-peaks achieved are related to each ionic species and were due to the RM-β-CD derivatives were formed with different number of methyl groups. Ions at m/z 1291.5, 1305.5, 1319.5, 1333.6, 1347.6, 1361.6, 1375.6, 1389.6 were according to eight derivatives bearing from eight to fifteen methyl substituents. The largest peaks for each degree of substitution corresponded to FA adducts. The average degree of substitution of the RM-β-CD was 12.2 per CD molecule calculated according to its abundance ratio.

The search of the ABZ:RM-β-CD inclusion complex was carried out in negative mode. Ions at m/z 1510.6, 1524.6, 1538.6, 1552.6, 1566.6, 1580.7, 1594.7 and 1608.7 corresponded to the complex in a molar ratio 1∶1, between ABZ (Ion at m/z 264.1) and the RM-β-CD with eight to fifteen methyl substituents. No strong peaks were detected above the 1608 m/z value, indicating the formation of an ABZ:RM-β-CD complex in 1∶1 and not 1∶2 molar ratio.

#### ROESY experiments

**Figure 4:**
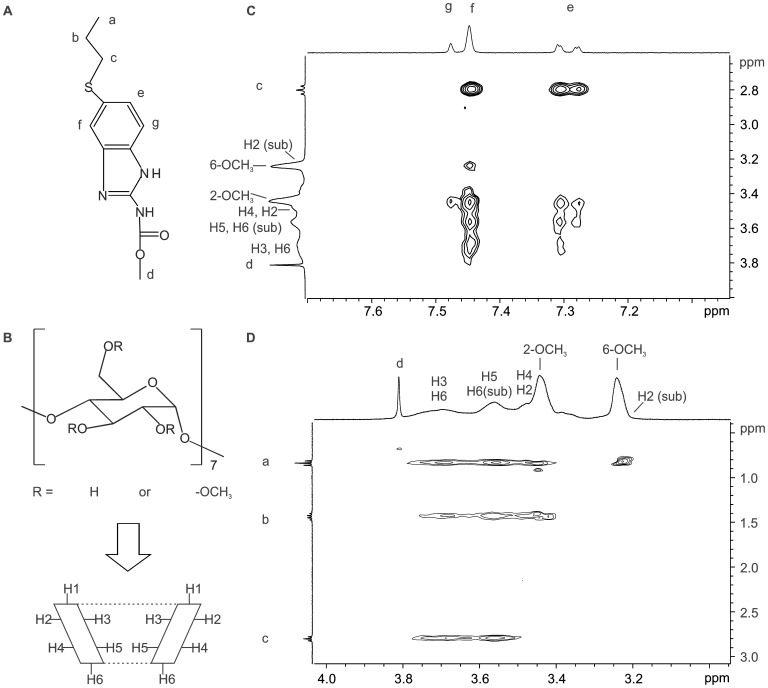
ROESY spectrum. A: ABZ proton labelling, B: RM-β-CD proton labelling. C and D: Plot of two dimensional ROESY spectrum of ABZ in the presence of RM-β-CD.

An unambiguous ^1^H NMR spectral assignment is required to establish the inclusion mode of ABZ in the RM-β-CD cavity. The labeling of all the signals in the ^1^H NMR spectra of ABZ and RM-β-CD were carried out using the information obtained from H–H COSY, H-C HSQC and H-C HMBC spectra (data not shown). The structure of both molecules is shown in Figure 4.

The changes in chemical shifts induced by direct methylation and the effects on nuclei next to a methylated site generated unresolved broad peaks in the ^1^H NMR spectrum. It could be observed that the signals of H2 and H6 of the substituted (sub) sites produced overlapping signals with the protons of 6-OCH_3_ and H5, respectively.

Figure 4 (C and D) shows a partial contour plot of a 2D ROESY spectrum of the inclusion complex ABZ:RM-β-CD. This spectrum exhibited intermolecular cross peaks between the aromatic ring protons (Figure 3 A: e, f and g) and RM-β-CD cavity protons (H3 and H5). These data suggest that the interactions of the aromatic ring take place in the CD cavity. Additionally, the presence of cross peaks between the protons of the methoxy groups of RM-β-CD and the ABZ protons a, b and f indicate that these drug protons were in a shallow position in the CD cavity.

#### Dissolution profiles

**Figure 5:**
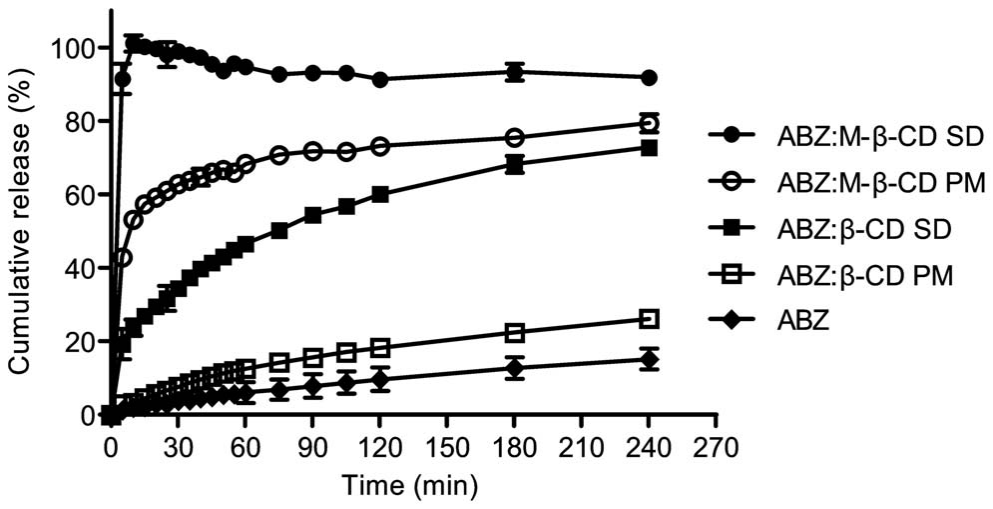
Dissolution profiles. Release profiles of ABZ (raw material), ABZ loaded in physical mixtures (β-CD and RM-β-CD), ABZ loaded in the inclusion complexes (spray drying) (β-CD and RM-β-CD). Test conditions: 0.1N HCl, 37 °C (n = 3±SD).

Dissolution profiles of ABZ:RM-β-CD, ABZ:β-CD, obtained by spray drying or physical mixtures, and non-treated ABZ were performed to determine the dissolution rate of ABZ loaded in each system (Figure 5). The profiles corresponding to ABZ:β-CD physical mixtures and the pure drug showed similar results, reaching only 26% and 15% drug release after six hours of the running assay. The ABZ:β-CD spray-dried complex and the physical mixtures prepared with ABZ:RM-β-CD achieved, respectively, 23% and 54% of drug release after 10 min of the running assay.

The dissolution profiles of ABZ loaded in the RM-β-CD inclusion complex prepared by spray drying exhibited a 100% drug release after 10 minutes of the running assay. The dissolution efficiency areas of ABZ:RM-β-CD physical mixtures (70.32%) and spray dried preparations (92.78%) were 7.6 and 10 times higher than the drug without any treatment (9.16%). These results demonstrate, undoubtedly, the improvement of the dissolution rate of ABZ loaded in this carrier.

### *In vivo* study of the antiparasitc efficacy of the ABZ:RM-β-CD complex

The spray dried inclusion complexes improved their intestinal anthelmintic efficacy compared with that of ABZ alone. As shown in Table 1, mice given the spray dried ABZ:RM-β-CD complex had the lowest mean parasitic burden as well as the lowest variance, and differed significantly from the non-treated control group. The ABZ-treated group increased its relative variability compared to the control mice as derived from the coefficients of variation. In contrast, the ABZ:RM-β-CD complex-treated group decreased its variability. These results support the notion that ABZ low solubility affects its bioavailability and induces a heterogeneous therapeutic response. While statistically significant differences between the treated groups were not seen, the effect of differences such as lower mean response and variability is consistent with the supposition of a better performance of the new formulation. The homogenous response observed in mice treated with the complex may be associated to a better dissolution rate, and potentially a better bioavailability of ABZ.

**Table 1:** Efficacy of the ABZ:RM-β-CD formulation on the muscle larval load of *Trichinella spiralis* infected CBi+ males.

Group	Relative larval load*	Coefficient of variation (%)	Larval load reduction percentage**
**I)ABZ**	580±133.4^a,b^	51.4	76^a^ (65–90)
**II)ABZ:RM-β-CD**	435±63.0^b^	32.4	84^a^ (79–90)
**III)Control**	2712±500.5^a^	41.3	–

Differences among groups were evaluated with the Kruskal-Wallis test, using Dunn’s post-test for comparisons between groups (**Relative larval load**), or by the non parametric Mann-Whitney test (**Larval load reduction percentage**).

For each variable, differences between groups not sharing the same superscript are significant at the 0.001 level.

Larval load was measured in the tongue, a preferred site of encystment in mice.

*Mean ± SEM **Median (range).

## Conclusions

The physicochemical evaluation of the inclusion complexes prepared with ABZ and RM-β-CD showed promising results. The drug release rate was remarkably improved; achieving dissolution efficiency areas 10 times higher than the drug alone demonstrating that the RM-β-CD is a suitable excipient for the design of oral dosage forms with poorly soluble compounds. Moreover, these results were consistent with the loss of crystallinity observed by XRD and DSC analysis. ABZ inclusion complex was also confirmed by NMR studies. The *in vivo* assessment of the complex antiparasitic effectiveness in a murine model of trichinellosis revealed a decrease in mice muscle parasitic burden compared with that of animals treated with the unmodified drug. Therefore, the inclusion complex with RM-β-CD by spray drying is an interesting alternative to treating parasitic intestinal infections.
